# Laparoscopic transabdominal preperitoneal repair versus open mesh plug repair for bilateral primary inguinal hernia

**DOI:** 10.1002/ags3.12314

**Published:** 2020-02-11

**Authors:** Yuichi Takayama, Yuji Kaneoka, Atsuyuki Maeda, Takamasa Takahashi, Masahito Uji

**Affiliations:** ^1^ Department of Surgery Ogaki Municipal Hospital Ogaki‐shi Japan

**Keywords:** bilateral inguinal hernia, laparoscopic transabdominal preperitoneal repair, open mesh plug repair

## Abstract

**Aim:**

A few studies comparing laparoscopic and open techniques have reported that open repair with mesh is the optimal operation for unilateral primary hernia. The aim of this study is to compare the outcomes of laparoscopic transabdominal preperitoneal repair (TAPP) versus open mesh plug repair (MP) for bilateral primary inguinal hernia.

**Methods:**

This was a retrospective study of 107 patients with bilateral primary inguinal hernia between January 2008 and December 2016. Of these patients, 49 underwent TAPP and 58 underwent MP. The surgical outcomes and the long‐term outcomes using a questionnaire were compared between TAPP and MP.

**Results:**

In the TAPP group, the operation time was significantly longer (103 vs 91 minutes; *P* = .019). The postoperative complication rate was not significantly different between the two groups. One patient (1.0%) in the TAPP group and five patients (4.3%) in the MP group suffered recurrence (*P* = .30). Postoperative groin pain was not significantly different (14% in the TAPP group vs 31% in the MP group; *P* = .065), but more patients required analgesics in the MP group (4.1% vs 17%; *P* = .036). The long‐term outcomes, according to a questionnaire, were not significantly different between the two groups. The median follow‐up period was 22 (range, 0.4‐52) months in the TAPP group and 40 (range, 0.5‐108) months in the MP group (*P* < .001).

**Conclusion:**

TAPP for bilateral primary inguinal hernia achieved better results than MP relative to postoperative pain and the use of medication for pain relief without increasing the complication and recurrence rates.

## Graphical abstract

This is the first report to compare laparoscopic transabdominal preperitoneal repair (TAPP) with open mesh plug repair (MP) for bilateral primary inguinal hernia. TAPP achieved better results than MP relative to postoperative pain and the use of medication for pain relief without increasing the complication and recurrence rates. The long‐term outcomes, according to a questionnaire, were not significantly different between the two groups.

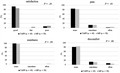

## 1 INTRODUCTION

Inguinal hernia repair is the most frequently performed operation in general surgery. Inguinal hernia repair has evolved from the old herniorrhaphy techniques to tension‐free repair using mesh and, ultimately, laparoscopic approaches. Laparoscopic inguinal hernia repair was first performed by Ger et al in 1988^1^ and has been proven as an efficient technique, serving a valuable alternative and offering the advantages of minimally invasive surgery.^2^ However, the optimal approach—open/anterior or laparoscopic/endoscopic/posterior repair—is still a topic of discussion.

In 2004, for unilateral primary inguinal hernia, a large Veterans’ Affairs study compared open mesh and laparoscopic techniques and declared that open repair with mesh was the optimal operation. This outcome linked the laparoscopic approach to increased complication and recurrence rates in addition to the need for general anesthesia.^3^ Over the last 12 years, multiple studies have compared these two operations and reported that the recurrence rates were similar, but they have shown conflicting results on the perioperative outcomes and costs.^4^, ^5^

For bilateral inguinal hernia, two prospective randomized trials^6^, ^7^ demonstrated that the laparoscopic technique compared with the open technique was associated with significantly less pain, significantly less need for analgesics, and significantly earlier resumption of work activities. Two large studies on bilateral inguinal hernia repair (2880 patients in 2010^8^ and 1336 patients in 2002^9^) showed that, compared to unilateral repair, bilateral TAPP was safe, comfortable for patients, and cost‐effective, without increased morbidity or recurrence risk. However, open inguinal hernia repair was performed with the Lichtenstein technique. Few studies have compared the laparoscopic technique with the mesh plug technique for bilateral inguinal hernia.

The aim of this study is to compare the outcomes of laparoscopic transabdominal preperitoneal repair (TAPP) versus open mesh plug repair (MP)^10^ for bilateral primary inguinal hernia based on a retrospective data review.

## 2 MATERIAL AND METHODS

This was a retrospective study of 107 patients with bilateral primary inguinal hernia between January 2008 and December 2016 in our institution. This study was approved by the Ethics Review Board of Ogaki Municipal Hospital. Of these patients, 49 underwent TAPP and 58 underwent MP. Since December 2012, TAPP has been performed. Before the introduction of TAPP, we performed open hernia repair in all patients. The inclusion criteria for TAPP were an age less than 80 years old and an Eastern Cooperative Oncology Group (ECOG) performance status of 0 or 1. The exclusion criteria were unstable angina or myocardial infarction, severe respiratory disease, and a history of previous lower abdominal surgery, except for appendectomy. Since December 2012, if patients were eligible for TAPP, the surgeon confirmed the diagnosis in the outpatient department and explained both procedures and written consent was obtained. According to our policy, a contralateral occult inguinal hernia identified at the time of TAPP repair is not repaired. All patients enrolled in this study were diagnosed with bilateral primary inguinal hernia prior to surgery.

All patients were given a single intravenous dose of antibiotics preoperatively. After surgery, analgesics were given on demand when the standard protocol did not achieve adequate pain control. If there are no signs of major complications, patients can be discharged on postoperative day (POD) 2 according to the perioperative and postoperative management protocols (clinical pathways). All patients were followed up as outpatients within 2‐3 weeks postoperatively to assess groin pain, medication requests, and any complications. A further clinical follow‐up was conducted by a patient's general practitioner, with referral back to the hospital if any problems developed.

The following variables were recorded from a retrospective review of medical records: clinical characteristics and surgical and postoperative outcomes (including complications). Postoperative complications such as seroma, bleeding, infection, orchitis, chronic pain, and recurrence were evaluated and were graded according to the Clavien–Dindo classification.^11^ Intra‐ and postoperative complications were recorded if a complication presented on at least one side. Seroma was defined as some amount of fluid collection in the inguinal region requiring puncture. Chronic pain was defined as the presence of pain 3 months after surgery that persists beyond 6 months after surgery.^12^ In addition, the parameters reflecting postoperative recovery, such as groin pain and analgesic use, were measured in the outpatient setting within 2‐3 weeks postoperatively.

In 2017, long‐term outcomes were analyzed retrospectively using a questionnaire. The questionnaire included six parts that covered: (a) the degree of satisfaction with the procedure (good, fair, poor); (b) pain (none, sometimes, often); (c) numbness (none, sometimes, often); (d) discomfort (none, sometimes, often); (e) recurrence (yes or no); and (f) the time required to return to normal activity. Patients who provided answers suggesting recurrence or hoping for a medical examination were asked to visit our department for confirmation.

### 2.1 Surgical techniques

#### 2.1.1 TAPP repair

The laparoscopic technique was performed using the transabdominal peritoneal route under general anesthesia without the placement of a nasogastric tube or urinary catheter. The 5‐mm optical trocar was placed at the upper rim of the umbilicus. Both working trocars, one 5‐mm and one 12‐mm trocar, were placed at the level of the navel to the right and the left of the border of the rectus abdominis. A mostly blunt dissection was performed strictly along the anatomical landmarks (rectus muscle, epigastric vessels, symphysis and Cooper's ligament, and transverse fascia) and ended in complete anatomical dissection of the whole pelvic floor. Thorough hemostasis should always be performed. Parietalization was especially important, which involves removing all adhesions between the retroperitoneal tissue (fascia spermatica) and the peritoneum down to the middle of the psoas muscle. Two 3D Max^®^ meshes (Bard Davol, Inc) were implanted, overlapping the defect. The mesh had to overlap the defect by at least 3 cm in each direction. The meshes were fixed into position using PermaFix^®^ (Bard Davol Inc) to the pectineal ligament and to the anterior abdominal wall. The peritoneum was closed with a 3‐0 Vicryl (polyglactin; Ethicon Inc) running suture. The skin was closed with a 4‐0 Vicryl (polyglactin; Ethicon Inc) interrupted suture.

#### 2.1.2 MP repair

Open inguinal hernia repair was carried out using the mesh plug technique under local anesthesia with sedation, if required.^10^ Through a 5‐8 cm skin incision, the external oblique aponeurosis was incised, and we carefully aimed to identify and preserve the ilio‐inguinal and ilio‐hypogastric nerves. For an indirect hernia, the hernia sac was completely freed from the cord structures and from the investing fibers of the transversalis fascia at the internal ring. A transversalis fascia and preperitoneal fascia exists at the internal ring and must be incised to enter the preperitoneal spaces. For a direct hernia, the attenuated transversalis fascia and preperitoneal fascia were circumcised and opened with electrocauterization along the demarcation of the herniated defect to enter the preperitoneal spaces. The peritoneum beneath the direct and indirect space was dissected from the transversalis fascia and preperitoneal fascia with the vas deferens, internal spermatic and deep inferior epigastric vessels parietalized; an adequate size of preperitoneal space was thus created. If these procedures are inadequate, recurrent herniation will occur. The inserted plug was placed below the internal ring or the opened direct space preperitoneally and fixed with three absorbable sutures. For both direct and indirect hernias, an onlay mesh was placed (unsutured) on the anterior surface of the posterior wall, with adequate coverage of pubis tubercle. After closure of the external oblique and Scarpa's fascia with an interrupted 3‐0 Vicryl (polyglactin; Ethicon Inc) suture, the skin was closed with a 4‐0 Vicryl (polyglactin; Ethicon Inc) running suture. The inserted plug and onlay mesh used were Perfix Plug^®^ (Bard Davol, Inc) (HWMP) until April 2010, and Light Perfix Plug^®^ (Bard Davol, Inc) (LWMP) after April 2010.

### 2.2 Statistical analysis

All statistical analyses were performed with EZR (Saitama Medical Centre, Jichi Medical University; Kanda, 2012), which is a graphical user interface for R (The R Foundation for Statistical Computing, version 2.13.0).^13^ To compare treatment groups, categorical variables were analyzed using the Chi‐squared test, and the Student's t‐test or the Mann–Whitney U test was used to compare continuous variables as appropriate. *P* values < .05 were considered statistically significant.

## 3 RESULTS

A total of 107 bilateral primary inguinal hernia patients, including 49 (98 hernias) TAPP and 58 (116 hernias) MP cases, were included in this study. Among MP cases, HWMP was used in 16 cases and LWMP in 42 cases. No conversion to an open procedure was recorded in the TAPP series. Clinical characteristics are summarized in Table 1. The median age of the MP group was significantly higher than that of the TAPP group, but there were no differences in gender and BMI between the two groups. The rates of comorbidities, cardiac angina, cerebrovascular disease, and COPD in the MP group were significantly higher than in the TAPP group. No patients had steroid therapy.

**Table 1:** Clinical characteristics and pathological findings of patients

	TAPP (n = 49)	MP (n = 58)	*P* value
Age, years (mean, range)	64.3 (22‐79)	72.4 (53‐96)	<.001
Sex (male)	47 (96)	54 (93)	.83
BMI, kg/m^2^ (mean, range)	22.3 (15.8‐28.4)	21.3 (16.6‐27.2)	.050
Comorbidities	19 (39)	38 (66)	.010
Hypertension	16 (32)	22 (38)	.71
Cardiac Angina	0	8 (14)	.020
Arrhythmia	2 (4.1)	5 (8.6)	.58
Diabetes	6 (12)	8 (14)	1
Cerebrovascular disease	1 (2.0)	8 (14)	.066
COPD	1(2.0)	6 (10)	.18
Previous laparotomy	9 (18)	15 (26)	.49

Note

Data are shown as number of patients (%).

Abbreviations: BMI, body mass index; COPD, chronic obstructive pulmonary disease.

Surgical outcomes are summarized in Table 2. In the TAPP group, the operation time was significantly longer (103 vs 91 minutes) and the estimated blood loss was significantly less (3.3 vs 9.3 mL). No intraoperative complications were observed in either group. Postoperative outcomes are presented in Table 3. The postoperative complication rate was not significantly different between the two groups. Seroma developed in five hernias (5.1%) in the TAPP group and in one hernia (0.9%) in the MP group (*P* = .15). Postoperative bleeding occurred in one hernia (0.9%) in the MP group. This patient had anti‐coagulant therapy for stable angina treatment. Subcutaneous hematoma was formed and was healed by astriction. A superficial wound infection occurred in one hernia (0.9%) in the MP group and was healed with conservative treatment; no mesh was removed. This patient had chronic obstructive pulmonary disease but no diabetes. Orchitis occurred in one hernia (1.0%) in the TAPP group and was healed with antibiotic treatment. No patients experienced chronic pain in either group. One patient (1.0%) in the TAPP group and five patients (4.3%) in the MP group suffered recurrence (*P* = .30). There was no postoperative mortality. Postoperative hospital stay was longer in the MP group. In the MP group, 15 patients (26%) stayed in the hospital for more than 3 days. The reasons were 12 cases of patient's choice, and one case each for fever, heparinization, and wound bleeding. Also, the rates of postoperative complications and recurrence did not significantly differ between the HWMP group and the LWMP group.

**Table 2:** Surgical outcomes

	TAPP (n = 49)	MP (n = 58)	*P* value
Operation time, min (mean, range)	102.9 (58‐198)	90.7 (48‐160)	.019
Blood Loss, mL (mean, range)	3.3 (1‐50)	9.3 (1‐75)	<.0032
Intraoperative complication	0	0	
Type of hernia (right side‐left side)			
Direct‐direct	18 (37)	23 (40)	.66
Indirect‐indirect	15 (31)	22 (38)	
Direct‐indirect	10 (20)	9 (16)	
Direct‐en pantalon	1 (2.0)	2 (3.4)	
Indirect‐en pantalon	4 (8.2)	1 (1.7)	
En pantalon‐en pantalon	1 (2.0)	1 (1.7)	

Note

Data are shown as number of patients (%).

**Table 3:** Postoperative outcomes

	TAPP (n = 49)	MP (n = 58)	*P* value
Complication^a^	7 (7.1)	8 (6.9)	1
Seroma^a^	5 (5.1) grade IIIa	1 (0.9) grade IIIa	.15
Bleeding^a^	0	1 (0.9) grade I	1
Wound infection^a^	0	1 (0.9) grade II	1
Orchitis^a^	1 (1.0) grade II	0	.93
Chronic pain	0	0	1
Recurrence^a^	1 (1.0) grade IIIb	5 (4.3) grade IIIb	.30
Postoperative hospital stay,days (median, range)	2 (2‐3)	2 (2‐10)	.017

Note

Data are shown as number of patients (%).Grade: according to Dindo‐Clavien classification.

a Adjusted for the number of hernias.

**Figure 1:**
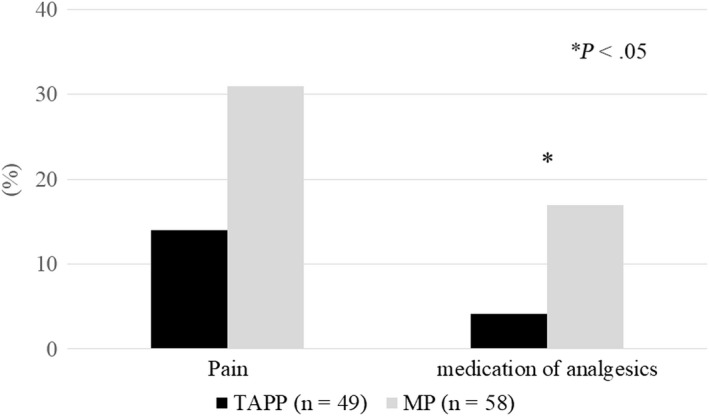
Postoperative pain and analgesic use for pain relief

The postoperative status of the patients at 2‐3 weeks after surgery is demonstrated in Figure 1. There was no significant difference in groin pain (14% in the TAPP group vs 31% in the MP group; *P* = .065), but more patients required analgesics in the MP group (4.1% vs 17%; *P* = .036). In addition, we divided the MP group into the HWMP group and the LWMP group, and compared the status between the three groups. The rates of groin pain and analgesics use for pain relief were lower in the TAPP group (14% and 4.1%) than in the HWMP group (25% and 19%) or the LWMP group (33% and 17%), though not statistically significant. The two MP groups had almost similar status.

**Figure 2:**
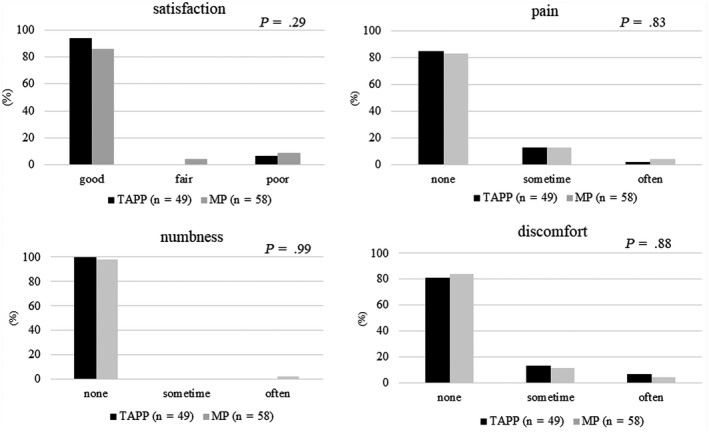
Long‐term outcomes using a questionnaire

Long‐term outcomes are demonstrated in Figure 2. Ten patients (TAPP, n = 1; MP, n = 9) died from unrelated causes. Questionnaires with answers were obtained from 93 patients (96.0%) [TAPP, n = 47 (97.9%); MP, n = 46 (93.9%)]. Eight patients (TAPP, n = 2; MP, n = 6) were assessed face‐to‐face, 13 patients (TAPP, n = 5; MP, n = 8) by telephone interview, and 72 patients (TAPP, n = 40; MP, n = 32) by mail. No patients with recurrence were observed from the questionnaires. No significant differences were observed between the two groups relative to satisfaction, pain, numbness, and discomfort. No patients complained of severe pain requiring treatment. The median number of days required to return to normal activity was not significantly different: 15 days (range, 1‐365) after TAPP and 20 days (range, 1‐180) after MP (*P* = .24).

The median follow‐up period was 22 (range, 0.4‐52) months in the TAPP group and 40 (range, 0.5‐108) months in the MP group (*P* < .001).

## 4 DISCUSSION

This study was designed to compare the clinical outcomes of TAPP and MP for bilateral primary inguinal hernia with particular attention to surgical, short‐term, and long‐term outcomes. In this study, TAPP procedure was performed under general anesthesia and MP procedure was performed under local anesthesia. Dimitrios et al compared prospective TAPP vs. MP repair performed under different anesthetic methods and showed that chronic pain incidence was lower for the TAPP group and there were no differences in other short‐ and long‐term complications.^14^ The anesthesia method does not seem to affect the postoperative outcomes. Our study showed that TAPP was associated with significantly longer operative time, but postoperative complications were similar in both groups. At 2‐3 weeks postoperatively, more patients in the MP group needed analgesics. TAPP was a feasible, safe, and less painful procedure.

The European Hernia Society guidelines recommend Lichtenstein and endoscopic procedure for unilateral hernia repair and, from a socioeconomic perspective, an endoscopic procedure for the active working population, especially for bilateral hernia repair.^15^ MP technique was developed in 1993.^10^ It is technically simple to learn and perform.^16^ In some studies, MP repair was superior to Lichtenstein repair regarding postoperative pain, quality of life of the patient, shorter duration of operation, and duration of hospital stay although the two methods were similar regarding both recurrence and complication rates.^17^, ^18^ The MP technique has been performed widely, especially in Japan.^19^ At our institution as well, the open procedure has been performed using the MP technique. A prospective study comparing TAPP and MP for unilateral inguinal hernia demonstrated that MP was faster, cheaper, technically easier, and resulted in fewer short‐ or long‐term complications and reduced the recurrence rate.^20^ Few studies have compared TAPP with MP for bilateral inguinal hernia. This is the first report to compare TAPP with MP for bilateral primary inguinal hernia.

Here, we summarized the data of the five studies comparing TAPP and the open technique for bilateral inguinal hernia^6^, ^7^, ^21^, ^22^ with respect to operation time, complications, recurrence, and time of disability (Table 4). The open procedures included preperitoneal prosthetic repair through a vertical midline incision in study 1, Shouldice repair in study 2, Lichtenstein repair in studies 3 and 4, and MP in our case. Overall, the duration of the operation (partial with a large variation) was slightly longer for TAPP than for the open procedure. The fairly long operative procedures were partly due to the lesser experience of the surgeons. TAPP was influenced by the learning curve. TAPP was introduced at our institution in December 2012 and we are inexperienced with regard to this technique. With a learning curve of 250 cases, TAPP could arguably be classified as a complex laparoscopic operation that requires additional training.^23^ If we continue to improve our laparoscopic skills, we believe that the operation time for TAPP will decrease. Although there are differences in the definitions of complications, our complication rates, except for recurrence (6.1% in the TAPP group and 2.6% in the MP group), were not higher than that of other studies and there were no differences between the two groups. A significant chronic complication of inguinal hernia repair is long‐term groin pain. Although initially reported as infrequent, this has recently been reported in up to 30% of patients undergoing open inguinal hernia repair.^24^ This number is significantly reduced with the laparoscopic approach. We evaluated chronic pain using medical records and a questionnaire and found that 2.1% of TAPP patients and 4.0% of MP patients reported “often” for pain and 13% of TAPP patients and 13% of MP patients reported “sometimes” for pain (Figure 2). No patients reported severe pain. Published recurrence rates for TAPP (under the auspices of randomized trials) vary between 0% and 15%. Published recurrence rates for open mesh repair (under the auspices of randomized trials) vary between 0% and 5%.^25^, ^26^ The comparative long‐term results at 5 years of randomized controlled trials between open and laparoscopic mesh repair of primary inguinal hernia show almost the same recurrence rate (open; 3%～5% vs laparoscopic; 2%～4%).^27^, ^28^ In our series, the recurrence rates for both TAPP (1.0%) and MP (4.3%) compared favorably with those previously published. Although some studies showed that most recurrences with mesh technique seem to appear early,^10^, ^29^, ^30^ it is necessary to follow‐up in the future because the follow‐up periods in our study are not long (median 22 months in the TAPP group and 44 months in the MP group). At reoperation in our recurrent case of TAPP, we noticed creasing of the mesh across the midline with medially located recurrences. We changed our practice and after an adequate extent of peritoneum was peeled away, mesh was placed, overlapping the defect by more than 3 cm, without creasing of the mesh. Since this change in practice, no recurrence has been observed in either unilateral or bilateral inguinal hernia repair cases. Regarding disability time, in our series, similar to other studies, the resumption of normal activity occurred earlier in the TAPP group, but without statistical significance. These results indicate that TAPP did not increase complication and recurrence rates and that the resumption of work activities occurred earlier, even with MP.

**Table 4:** Summary of data collected from literature concerning surgical outcomes compared open and TAPP for bilateral inguinal hernia

Study	Study design	Year	n		Operation time (min)		Complication^a^ (%)		Recurrence^a^ (%)		Time of disability (days)	
			Open (procedure)	TAPP	Open	TAPP	Open	TAPP	Open	TAPP	Open	TAPP
1	Prospective^16^	1996	47 (preperitoneal prosthetic repair)	25	69	86	33	48	4.5	3	22	9
2	Retrospective^17^	1998	39 (Shouldice)	39	121	140	3.8	7.6	5.1	1.3	56	18
3	RCT^6^	2001	23 (Lichtenstein)	20	99	95	18	17	2.1	0	30	16
4	RCT^7^	2003	60 (Lichtenstein)	59	40	55	18	11	0.8	3.4	42	11
Present	Retrospective	2017	58 (MP)	49	91	103	2.6	6.1	4.3	1	20	15

Abbreviation: RCT, Randomized Control Trial.

a Adjusted for the number of hernias Complication; except for Recurrence.

We evaluated postoperative pain in the outpatient setting within 2‐3 weeks postoperatively. Postoperative pain during the first few days after surgery can be affected by anesthesia (general or local).^14^ In the MP group, the rate of groin pain was greater (14% vs 31%), though not statistically significant, and more patients required analgesics (4.1% vs 17%; *P* = .036). The results of Pikoulis et al demonstrated that the incidence of postoperative pain was equal between the MP group and the TAPP group for unilateral inguinal hernia repair. It seems logical that benefits such as less postoperative pain were evident in patients with bilateral inguinal hernia undergoing TAPP since an open approach requires two incisions.

The long‐term complications that we focused on were recurrence, chronic pain, numbness, and discomfort. Finally, we asked the patients to report their satisfaction with the procedure. The retrospective analysis of 216 patients who underwent open or laparoscopic repair using a Short‐Form 36 (SF‐36) revealed no significant difference scores between the two groups.^31^ Additionally, there were no significant differences in recurrence, pain, numbness, and discomfort between the two groups in our study, indicating that satisfaction with the procedure was similar in both groups and was comparable to previously reported results.^32^

The present study has several limitations. The main limitation includes the possible presence of biases, including age and comorbidities, because of the retrospective study design. The sample size was not large (49 cases in the TAPP group vs 58 cases in the MP group) and the follow‐up periods were not long (median 22 months in the TAPP group and 44 months in the MP group). However, the results are helpful in terms of processing prospective randomized clinical trials comparing TAPP and MP for bilateral inguinal hernia repair.

In conclusion, TAPP for bilateral primary inguinal hernia achieved better results than MP relative to postoperative pain and the use of medication for pain relief without increasing complication and recurrence rates.

## DISCLOSURE

Conflict of Interest: Authors declare no conflict of interests for this article.

## Ethical Approval

The protocol for this research project has been approved by the Ethics Review Board of Ogaki Municipal Hospital. and it conforms to the provisions of the Declaration of Helsinki. Committee of Ogaki Municipal Hospital, Approval No. 20161222‐8. Informed consent was obtained from all the patients.

## References

[1] Ger R , Monroe K , Duvivier R , Mishrick A . Management of indirect inguinal hernias by laparoscopic closure of the neck of the sac. Am J Surg. 1990;159:370–3. 2138432 10.1016/s0002-9610(05)81273-5

[2] Tadaki C , Lomelin D , Simorov A , Jones R , Humphreys M , da Silva M , et al. Perioperative outcomes and costs of laparoscopic versus open inguinal hernia repair. Hernia. 2016;20:399–404. 26874507 10.1007/s10029-016-1465-y

[3] Neumayer L , Giobbie‐Hurder A , Jonasson O , Fitzgibbons R , Dunlop D , Gibbs J , et al. Veterans affairs cooperative studies program I. Open mesh versus laparoscopic mesh repair of inguinal hernia. N Engl J Med. 2004;350:1819–27. 15107485 10.1056/NEJMoa040093

[4] Pokorny H , Klingler A , Schmid T , Fortelny R , Hollinsky C , Kawji R , et al. Recurrence and complications after laparoscopic versus open inguinal hernia repair: results of a prospective randomized multicenter trial. Hernia. 2008;12:385–9. 18283518 10.1007/s10029-008-0357-1

[5] Dhankhar DS , Sharma N , Mishra T , Kaur N , Singh S , Gupta S . Totally extraperitoneal repair under general anesthesia versus Lichtenstein repair under local anesthesia for unilateral inguinal hernia: a prospective randomized controlled trial. Surg Endosc. 2014;28:996–1002. 24196555 10.1007/s00464-013-3269-9

[6] Sarli L , Iusco DR , Sansebastiano G , Costi R . Simultaneous repair of bilateral inguinal hernias: a prospective, randomized study of open, tension‐free versus laparoscopic approach. Surg Laparosc Endosc Percutan Tech. 2001;11:262–7. 11525372 10.1097/00129689-200108000-00007

[7] Mahon D , Decadt B , Rhodes M . Prospective randomized trial of laparoscopic (transabdominal preperitoneal) vs open (mesh) repair for bilateral and recurrent inguinal hernia. Surg Endosc. 2003;17:1386–90. 12802653 10.1007/s00464-002-9223-x

[8] Wauschkuhn CA , Schwarz J , Boekeler U , Bittner R . Laparoscopic inguinal hernia repair: gold standard in bilateral hernia repair? Results of more than 2800 patients in comparison to literature. Surg Endosc. 2010;24:3026–30. 20454807 10.1007/s00464-010-1079-x

[9] Schmedt CG , Daubler P , Leibl BJ , Kraft K , Bittner R . Laparoscopic Hernia Repair Study T. Simultaneous bilateral laparoscopic inguinal hernia repair: an analysis of 1336 consecutive cases at a single center. Surg Endosc. 2002;16:240–4. 11967671 10.1007/s00464-001-8184-9

[10] Rutkow IM , Robbins AW . "Tension‐free" inguinal herniorrhaphy: a preliminary report on the "mesh plug" technique. Surgery. 1993;114:3–8. 8356522

[11] Dindo D , Demartines N , Clavien PA . Classification of surgical complications: a new proposal with evaluation in a cohort of 6336 patients and results of a survey. Ann Surg. 2004;240:205–13. 15273542 10.1097/01.sla.0000133083.54934.ae PMC1360123

[12] Alfieri S , Amid PK , Campanelli G , Izard G , Kehlet H , Wijsmuller AR , et al. International guidelines for prevention and management of post‐operative chronic pain following inguinal hernia surgery. Hernia. 2011;15:239–49. 21365287 10.1007/s10029-011-0798-9

[13] Kanda Y . Investigation of the freely available easy‐to‐use software 'EZR' for medical statistics. Bone Marrow Transplant. 2013;48:452–8. 23208313 10.1038/bmt.2012.244 PMC3590441

[14] Symeonidis D , Baloyiannis I , Koukoulis G , Pratsas K , Georgopoulou S , Efthymiou M , et al. Prospective non‐randomized comparison of open versus laparoscopic transabdominal preperitoneal (TAPP) inguinal hernia repair under different anesthetic methods. Surg Today. 2014;44:906–13. 24318366 10.1007/s00595-013-0805-0

[15] Simons MP , Aufenacker T , Bay‐Nielsen M , Bouillot JL , Campanelli G , Conze J , et al. European Hernia Society guidelines on the treatment of inguinal hernia in adult patients. Hernia. 2009;13:343–403. 19636493 10.1007/s10029-009-0529-7 PMC2719730

[16] Gilbert AI , Graham MF . Sutureless technique: second version. Can J Surg. 1997;40:209–12. 9194782 PMC3952998

[17] Destek S , Gul VO . Comparison of Lichtenstein Repair and Mesh Plug Repair Methods in The Treatment of Indirect Inguinal Hernia. Cureus. 2018;10:e2935. 30202667 10.7759/cureus.2935 PMC6128592

[18] Fasih T , Mahapatra TK , Waddington RT . Early results of inguinal hernia repair by the 'mesh plug' technique–first 200 cases. Ann R Coll Surg Engl. 2000;82:396–400. 11103156 PMC2503476

[19] Negro P , D'Amore L , Gossetti F . Lichtenstein's operation, mesh plug, or prolene hernia system repair for groin hernia: which is better? Ann Surg 2010;252:199–200. 20562601 10.1097/SLA.0b013e3181e48743

[20] Pikoulis E , Tsigris C , Diamantis T , Delis S , Tsatsoulis P , Georgopoulos S , et al. Laparoscopic preperitoneal mesh repair or tension‐free mesh plug technique? A prospective study of 471 patients with 543 inguinal hernias. Eur J Surg. 2002;168:587–91. 12699093 10.1080/11024150201680003

[21] Velasco JM , Gelman C , Vallina VL . Preperitoneal bilateral inguinal herniorrhaphy evolution of a technique from conventional to laparoscopic. Surg Endosc. 1996;10:122–7. 8932612 10.1007/s004649910029

[22] Krahenbuhl L , Schafer M , Schilling M , Kuzinkovas V , Buchler MW . Simultaneous repair of bilateral groin hernias: open or laparoscopic approach? Surg Laparosc Endosc. 1998;8:313–8. 9703609

[23] Neumayer LA , Gawande AA , Wang J , Giobbie‐Hurder A , Itani KM , Fitzgibbons RJ Jr , et al. Investigators CSP. Proficiency of surgeons in inguinal hernia repair: effect of experience and age. Ann Surg. 2005;242:344–8; discussion 348–352. 16135920 10.1097/01.sla.0000179644.02187.ea PMC1357742

[24] Poobalan AS , Bruce J , King PM , Chambers WA , Krukowski ZH , Smith WC . Chronic pain and quality of life following open inguinal hernia repair. Br J Surg. 2001;88:1122–6. 11488800 10.1046/j.0007-1323.2001.01828.x

[25] Butters M , Redecke J , Koninger J . Long‐term results of a randomized clinical trial of Shouldice, Lichtenstein and transabdominal preperitoneal hernia repairs. Br J Surg. 2007;94:562–5. 17443855 10.1002/bjs.5733

[26] Bittner R , Sauerland S , Schmedt CG . Comparison of endoscopic techniques vs Shouldice and other open nonmesh techniques for inguinal hernia repair: a meta‐analysis of randomized controlled trials. Surg Endosc. 2005;19:605–15. 15789255 10.1007/s00464-004-9049-9

[27] Hallen M , Bergenfelz A , Westerdahl J . Laparoscopic extraperitoneal inguinal hernia repair versus open mesh repair: long‐term follow‐up of a randomized controlled trial. Surgery. 2008;143:313–7. 18291251 10.1016/j.surg.2007.09.028

[28] O'Dwyer PJ . Current status of the debate on laparoscopic hernia repair. Br Med Bull. 2004;70:105–18. 15509718 10.1093/bmb/ldh027

[29] Rutkow IM , Robbins AW . The mesh plug technique for recurrent groin herniorrhaphy: a nine‐year experience of 407 repairs. Surgery. 1998;124:844–7. 9823397

[30] Zieren J , Hoksch B , Wenger FA , Opitz I , Muller JM . Inguinal hernia repair in the new millennium: plug and patch repair with local anesthesia. World J Surg. 2001;25:138–41. 11338012 10.1007/s002680020093

[31] Srsen D , Druzijanic N , Pogorelic Z , Perko Z , Juricić J , Kraljević D , et al. Quality of life analysis after open and laparoscopic inguinal hernia repair–retrospective study. Hepatogastroenterology. 2008;55:2112–5. 19260487

[32] Schrenk P , Woisetschlager R , Rieger R , Wayand W . Prospective randomized trial comparing postoperative pain and return to physical activity after transabdominal preperitoneal, total preperitoneal or Shouldice technique for inguinal hernia repair. Br J Surg. 1996;83:1563–6. 9014675 10.1002/bjs.1800831124

